# FITC Conjugation Markedly Enhances Hepatic Clearance of N-Formyl Peptides

**DOI:** 10.1371/journal.pone.0160602

**Published:** 2016-08-05

**Authors:** Cristina Ionica Øie, Igor Snapkov, Kjetil Elvevold, Baldur Sveinbjørnsson, Bård Smedsrød

**Affiliations:** 1 Vascular Biology Research Group, Department of Medical Biology, Faculty of Health Sciences, University of Tromsø, Tromsø, Norway; 2 Molecular Inflammation Research Group, Department of Medical Biology, Faculty of Health Sciences, University of Tromsø, Tromsø, Norway; 3 D’Liver AS, Forskningsparken, Tromsø, Norway; 4 Childhood Cancer Research Unit, Department of Women's and Children's Health, Karolinska Institutet, Astrid Lindgren Children's Hospital, Stockholm, Sweden; Hospital Clinic de Barcelona, SPAIN

## Abstract

In both septic and aseptic inflammation, N-formyl peptides may enter the circulation and induce a systemic inflammatory response syndrome similar to that observed during septic shock. The inflammatory response is brought about by the binding of N-formyl peptide to formyl peptide receptors (FPRs), specific signaling receptors expressed on myeloid as well as non-myeloid cells involved in the inflammatory process. N-formyl peptides conjugated with fluorochromes, such as fluorescein isothiocyanate (FITC) are increasingly experimentally used to identify tissues involved in inflammation. Hypothesizing that the process of FITC-conjugation may transfer formyl peptide to a ligand that is efficiently cleared from the circulation by the natural powerful hepatic scavenging regime we studied the biodistribution of intravenously administered FITC-fNLPNTL (Fluorescein-isothiocyanate- N-Formyl-Nle-Leu-Phe-Nle-Tyr-Lys) in mice. Our findings can be summarized as follows: i) In contrast to unconjugated fNLPNTL, FITC-fNLPNTL was rapidly taken up in the liver; ii) Mouse and human liver sinusoidal endothelial cells (LSECs) and hepatocytes express formyl peptide receptor 1 (FRP1) on both mRNA (PCR) and protein (Western blot) levels; iii) Immunohistochemistry showed that mouse and human liver sections expressed FRP1 in LSECs and hepatocytes; and iv) Uptake of FITC-fNLPNTL could be largely blocked in mouse and human hepatocytes by surplus-unconjugated fNLPNTL, thereby suggesting that the hepatocytes in both species recognized FITC-fNLPNTL and fNLPNTL as indistinguishable ligands. This was in contrast to the mouse and human LSECs, in which the uptake of FITC-fNLPNTL was mediated by both FRP1 and a scavenger receptor, specifically expressed on LSECs. Based on these results we conclude that a significant proportion of FITC-fNLPNTL is taken up in LSECs via a scavenger receptor naturally expressed in these cells. This calls for great caution when using FITC-fNLPNTL and other chromogen-conjugated formyl peptides as a probe to identify cells in a liver engaged in inflammation. Moreover, our finding emphasizes the role of the liver as an important neutralizer of otherwise strong inflammatory signals such as formyl peptides.

## Introduction

To maintain homeostasis, the animal body is equipped with a powerful system of hepatic scavenger cells that remove circulating waste that otherwise might represent potential harm to the cells of the body. In this context, waste represents foreign stimuli such as microbial-derived macromolecules but also endogenous molecules that are released into circulation due to cell death or cell injury.

Innate immune responses in liver cells rely on the expression of pattern recognition receptors (PRRs) that recognize pathogen-associated molecular patterns, PAMPs, or endogenous molecules that are able to cause damage, referred to as damage-associated molecular patterns, DAMPs [1, 2]. The PAMPs are conserved molecular motifs found in microbial pathogens, which include viral nucleic acids and various bacteria-derived molecules. On the other hand, DAMPs represent non-microbial danger signals composed of host molecules often released by necrotic cells in the context of tissue damage. Both PAMPS and DAMPS trigger innate immune signaling and promote inflammation [2, 3].

Several types of PRRs are expressed in the liver. Most important are the endocytic mannose receptor and stabilins present on liver sinusoidal endothelial cells (LSECs) [1], which are utilized by LSECs to remove an array of circulating PAMPs and DAMPs. The LSEC expresses other important PRRs, including several toll-like receptors [4–6].

Mitochondria are organelles thought to arise from a symbiotic relationship with a host cell. Thus, the release of mitochondrial components such as DNA or N-formyl peptides may trigger strong inflammatory responses. Upon bacterial infection, as well as tissue injury, N-formyl peptides may enter the circulation and cause induction of systemic inflammatory response syndrome similar to that observed during septic shock [7, 8].

Therefore, it is of the utmost importance that these potentially dangerous macromolecules be efficiently removed from the blood circulation.

N-formyl peptide acts as typical PAMPs/DAMPs that can attract leukocytes to the sites of infection or tissue damage [9]. Formyl peptide receptor 1 (FPR1) is a cell surface PRR that binds and is activated by N-formyl-peptides originally described in leukocytes, which are important for the induction of inflammation and immune cell activation. More recently, FPR1 has also been detected in cells of non-myeloid origin such as glial cells, endocrine cells of the thyroid and adrenal glands, smooth muscle cells and lens epithelial cells, hence indicating the involvement of the receptor in a wide variety of both inflammatory and immunological responses [10, 11].

Intravenously administered formyl peptides conjugated with fluorescein isothiocyanate (FITC) or other fluorochrome are increasingly used as a probe to identify cells and tissue involved in inflammation [12–15]. The rationale for this is that fluorochrome-labeled formyl peptides will target cells that express FPR1. Knowing that conjugation with FITC increases the hepatic clearance of biomolecules [16] we found it pertinent to determine to what extent conjugation with FITC transform formyl peptide into a ligand for the natural hepatic clearance mechanisms.

**Fig 1:**
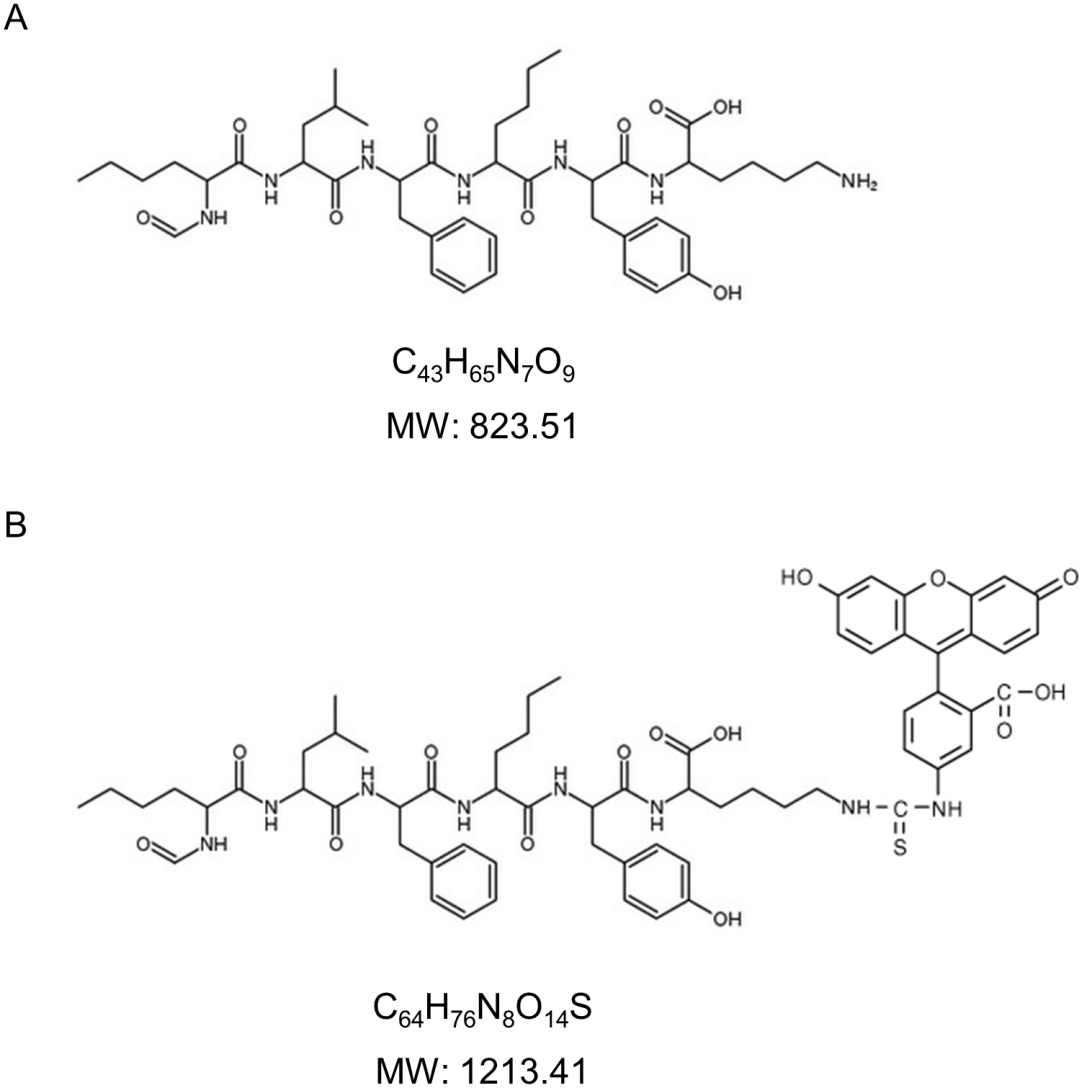
Structure of the peptides used in the study. Chemical structure and molecular mass of fNLPNTL (A) and FITC-fNLPNTL (B).

For this reason, in the present study we chose to use N-Formyl-Nle-Leu-Phe-Nle-Tyr-Lys (fNLPNTL) (Fig 1) and found that blood borne FITC-fNLPNTL, but not unconjugated fNLPNTL is rapidly taken up in mouse liver, with a high uptake in both isolated LSECs and liver parenchymal cells (hepatocytes). In addition, studies were carried out with human liver and isolated human LSECs and hepatocytes to determine to what extent the findings with the mouse model translated to human liver and liver cells. Our results show that intravenously administered FITC-fNLPNTL is taken up to a great extent in normal liver and liver cells via natural LSEC-specific scavenger receptors, and hence must be used with great caution if the purpose is to identify sites of inflammation.

## Materials and Methods

### Reagents

Collagenase was from Worthington Biochemical Corporation (Lakewood, NJ), and Liberase^™^ (collagenase) from Roche Applied Science (Oslo, Norway). Iodogen^™^ was from Pierce Chemicals (Rockford, IL), and carrier-free Na^125^I from PerkinElmer Norge AS (Oslo, Norway). Bovine serum albumin was from MP Biomedicals (Solon, OH), and bovine collagen type I (Vitrogen 100) from Cohesion Technologies (Palo Alto, CA). Human fibronectin purified from human plasma by affinity chromatography on Gelatin Sepharose 4B was a kind gift from Dr. Peter McCourt. Percoll^™^ and PD-10 columns (Sephadex G-25) were purchased from GE Healthcare (Uppsala, Sweden). Roswell Park Memorial Institute (RPMI) 1640 cell culture medium (supplemented with 20 mM sodium bicarbonate, 0.006% penicillin, and 0.01% streptomycin) was from Sigma-Aldrich (St Louis, MO). Human endothelial serum-free basal medium (HESFM) was obtained from Life Technologies. Vascular endothelial growth factor (VEGF) and hepatocyte growth factor (HGF) were from Peprotech, Germany. Falcon cell culture plates were from BD Biosciences (San Jose, CA), and Permanox chamber slides from VWR, Norway. N-Formyl-Nle-Leu-Phe-Nle-Tyr-Lys (fNLPNTL) was purchased from Phoenix Pharmaceuticals (Burlingame, CA, USA), and its fluorescein isothiocyanate derivative (FITC-fNLPNTL) from Thermo Scientific (Oslo, Norway).

Formaldehyde-treated bovine serum albumin (FSA) was prepared as described [17].

Polyclonal rabbit anti-FPR1 (H-230-Santa Cruz Biotech), rat-anti mouse CD206, clone MCA2235 (AbD Serotec, Oxford, UK), goat anti-human CD206 (R&D Systems), Alexa Fluor^®^-488 and -594 conjugated secondary antibodies (InVitrogen), and Draq5 (BioStatus Limited, Leicestershire, UK) were used for immunolabeling of cells and tissue.

### Ethics Statement

All animal experiments were approved by the Norwegian National Animal Research Authority (NARA) (approval IDs: ID-4689; ID-6688). The substances used for mouse anesthesia were a combination of tiletamine hydrochloride (Zoletil forte vet, Virbac, Norway), xylazine (Rompun, Bayer Nordic, Norway) and fentanyl (Actavis, Norway) (ZRF).

Human liver tissue was obtained from patients undergoing hepatic resections for colorectal cancer carried out at The National Hospital, Oslo, Norway. The patients participating in this study provided their signed consent after reading the information form. These signed documents were brought to our laboratory along with the liver resections from each individual patient who had agreed to participate. Same documents are filed along with other lab protocol documents in our laboratory. The Regional Committee for Medical and Health Research Ethics in North Norway approved this consent procedure (reference number 2011/686).

### Animals

Wild-type C57BL/6 male mice were obtained from the Charles River Laboratory, France. The animals were housed in rooms specially designed for mice, with 12 h/12 h day-night cycle, and free access to water and food (standard chow, Scanbur BK, Nittedal, Norway).

### Labeling procedures

Unconjugated fNLPNTL and FITC- fNLPNTL were reconstituted at a concentration of 4 mg/ml in DMSO. Twenty μl of the solution, corresponding to 100 μg of each peptide was further diluted in PBS to a final volume of 200 μl and incubated with carrier-free Na^125^I for 1h at RT, using Iodogen as described by the manufacturer. The labeled peptides were separated from unbound ^125^I by elution with PBS through PD-10 columns. The specific radioactivities were 0.98 x 10^6^ cpm/μg and 0.12 x 10^6^ cpm/μg for ^125^I-FITC-fNLPNTL and ^125^I-fNLPNTL, respectively.

### Anatomical distribution of radiolabeled peptides

Prior to experiments, mice were weighed and pre-warmed in a temperature controlled chamber to stimulate dilation of the tail veins. Trace amounts of ^125^I-FITC-fNLPNTL or ^125^I-fNLPNTL (8 μg in 137 μl PBS) were i.v. injected in non-anesthetized mice. The mice were anesthetized immediately after the i.v. injection by intraperitoneally injection of the ZRF mixture, followed by another intraperitoneal injection of a 1/3 dose of ZRF, 20 min later. Thirty min after i.v. administration of the peptides, a blood sample of 5 μl was collected from the tip of the tail using pre-calibrated glass pipettes. The abdominal cavity was opened and the body was cleared of blood and free radio-tracer from the vasculature by systemic perfusion through the heart with PBS. Internal organs and parts of the body were collected in pre-weighed test tubes: liver, spleen, kidneys, stomach, intestines, urinary bladder including urine, lungs, heart, muscle, head, tail, and the rest of the carcass. The amount of radioactivity was measured in all organs and in the blood sample. The radioactivity per total volume of blood was calculated considering that the average amount of blood in a mouse is approximately 58.5 ml per kg of bodyweight (according to the National Centre for the Replacement Refinement & Reduction of Animals in Research). The amount of tracer recovered was the sum of radioactivity of individual organs, carcass and the radioactivity calculated in total blood volume.

### Hepatocytes (Heps) and sinusoidal endothelial cells (LSECs) isolation from mouse and human liver

Mouse Heps (mHeps) and LSECs (mLSECs) were prepared by collagenase (Liberase) perfusion of mouse livers, followed by a low-speed differential centrifugation to separate Heps, and density sedimentation of the non-Heps enriched in the supernatant, on 25%– 45% Percoll gradients to collect LSECs and Kupffer cells (KCs), as described [18]. Mouse LSECs were obtained after a panning step of 8 min at 37°C to remove KCs. The purity of mLSECs was between 90 and 95% as judged by fenestration assessed by scanning electron microscopy and positive immunostaining for the LSEC specific marker, stabilin 2. The remaining 5–10% of cells in these cultures represented KCs/monocytes and stellate cells.

Human hepatocytes (hHeps) and LSECs (hLSECs) were isolated from encapsulated liver resections based on a method described elsewhere [19]. The procedure involves collagenase perfusion through cut blood vessels exposed on the surface of the liver resection. hHeps were isolated by low speed differential centrifugation, and the hLSECs purified by immunomagnetic isolation using mouse anti-human CD32 (Fitzgeralg Industries International INC, North Acton, MA, USA) and Dynabeads^®^ Sheep anti-mouse IgG (Thermo Scientific, Oslo, Norway). The viability of hLSECs as judged by trypan blue exclusion was 95%, and the yield of hLSECs varied from 1 to 10 million depending on the size of the liver resection. The purity of the hLSECs isolation was between 85 and 95%, as judged by light microscopy. This variation in purity is due to donor variation.

Primary mouse and human liver cell cultures were established on glass cover slides pre-coated with collagen type I and human fibronectin, respectively. Heps cultures were maintained in William’s E supplemented with antibiotics, glutamine, HEPES, insulin and dexamethasone, mLSECs in serum-free RPMI-1640 medium, and hLSECs in HESFM supplemented with antibiotics, 10% FCS, 10 ng/ml VEGF and 10 ng/ml HGF. Cultures of Heps and LSECs were incubated in 20% O_2_ or 5% O_2_, respectively.

### Immunohistochemistry

Liver tissue was embedded in Tissue-Tek OCT Compound (Sakura Finetek, Staufen, Germany), snap-frozen in liquid nitrogen, cut on a cryostat and stored at -80°C until use.

Sections were fixed with cold acetone for 10 min, washed 3X in PBS and then incubated with anti-FPR1 antibody. For co-localization immunostaining, mouse and human liver tissue sections were incubated overnight at 4°C with a primary anti-CD206 (Mannose receptor) antibody for the identification of LSECs and with anti-FPR1 antibody. For fluorescence visualization, anti-rabbit Alexa Fluor^®^-488, anti-rat Alexa Fluor^®^-594 or anti-goat Alexa Fluor^®^-594 were used. A matched isotype was also used as a control for nonspecific background staining. Images were taken on Zeiss LSM 780 microscope using the same acquisition settings for all sections.

### Immunocytochemistry

Primary cultures of mouse and human Heps and LSECs on Permanox chamber slides coated with human fibronectin were starved by 4 h of pre-incubation in a serum-free culture medium, and then challenged with 20 μg/ml FITC-fNLPNTL in serum-free culture medium for 1h at 37°C. The cells were extensively washed with PBS to remove unbound ligands and fixed with 4% PFA for 30 min prior to immunostaining. Non-specific binding was blocked by incubating the cells for 60 min at RT with 1% bovine serum albumin (BSA) in Tris-buffered saline with Tween (TBST). This was followed by 1h of incubation at 37°C with primary antibody (1:200) and then washed with TBST and incubated with Alexa Fluor^®^-488 conjugated goat anti-rabbit (1:1000) for 60 min at RT. For negative controls, isotype IgGs (same concentration as primary antibodies) or secondary antibodies only were used instead of the primary antibody. Nuclei were stained with a solution of 1:1000 Draq5 in PBS. The liver sections were then mounted with Dako Fluorescent Mounting Medium (Dako) and covered with glass coverslips. Images were taken using a confocal laser scanning microscopy (Zeiss LSM 780 microscope; Zeiss, Germany).

### Endocytosis competition of FITC-fNLPNTL by mouse and human Heps and LSECs

Primary cultures of mouse and human Heps and LSECs were seeded on Permanox chamber slides coated with human fibronectin. Following 4 h of serum-free starvation, the cells were incubated for 60 min at 37°C with 20 μg/ml of FITC-fNLPNTL in a serum-free culture medium, in the presence or absence of 100 μg/ml of native, non-FITC, fNLPNTL and FSA. The cells were pre-incubated with fNLPNTL or FSA for 30 min prior to the addition of the FITC-fNLPNTL. After removal of unbound ligand by extensive washing with PBS, the cultures were fixed with 4% PFA and processed for fluorescence microscopy as described above.

### RNA isolation

Total RNA was isolated from pelleted cells and whole tissue using the Qiagen RNeasy Mini Kit (QiagenHilden, Germany) according to the manufacturer's protocol. The quantity and quality of the extracted RNA was determined by the use of a spectrophotometer NanoDrop ND-1000 (Wilmington, DE, USA). cDNA was synthesized from 1 μg of total RNA using the QuantiTect Reverse Transcription Kit (Qiagen) according to the manufacturer's protocol.

### Quantitative PCR assay

PCR was performed in a 25 μl reaction mixture containing 2 μl of cDNA (from isolated RNA), 12.5 μl of the JumpStart REDTaq^®^ Ready Mix (Sigma-Aldrich), 0.5 μl of 10 μM forward and reverse primers, and 9.5 μl of ddH_2_O. The reactions were performed in a BioRad T-100 thermal cycler with the following conditions: initial denaturation at 95°C for 2 min, denaturation at 95°C for 30 sec, annealing at 58°C for 30 sec and extension at 72°C for 2 min. In total, 35 cycles were conducted with a final extension at 72°C for 10 min. PCR products were electrophoresed on 2% agarose gel and visualized under UV light. The sequences of PCR primers were human FPR1 forward: 5’-TGCCCTGGCCTTCTTCAACAGC-3’; and reverse: 5’-CGGGAAGGGCGTGGATCAGC-3’(PrimerBank ID 36951095c1, http://pga.mgh.harvard.edu/primerbank/index.html); human STAB2 forward: 5’-GTGCCCGGATGGTTACACC-3’; and reverse: 5’-CTTCCTACAAATATGGCGGCAT-3’ (PrimerBank ID 61743979c1); human CRP forward: 5’-TCGTATGCCACCAAGAGACAAGACA-3’; and reverse: 5’-AACACTTCGCCTTGCACTTCATACT-3’; human β-actin forward: 5’-CTCGACACCAGGGCGTTAT-3’; and reverse: 5’-CCACTCCATGCTCGATAGGAT-3’; mouse FPR1 forward: 5’-ACAGCCTGTACTTTCGAC-3’; and reverse: 5’-CTGGAAGTTAGAGCCCGTTC-3’; mouse STAB2 forward: 5’-AGCTGCTGCCTTTAATCCTCA-3’; and reverse: 5’-ACTCCGTCTTGATGGTTAGAGTA-3’ (PrimerBank ID 20149764a1); mouse GAPDH forward: 5’- ACCACAGTCCATGCCATCAC -3’; and reverse: 5’- TCCACCACCCTGTTGCTGTA -3’.

### Western blot analysis

Homogenized tissue specimens and cells were lysed directly in a RIPA lysis buffer (Pierce Biotechnology, Rockford, IL, USA) with both a protease inhibitor cocktail (Roche Applied Science, Basel, Switzerland) and Halt phosphatase inhibitor cocktail (Pierce Biotechnology). A mixture containing NuPAGE LDS Sample Buffer, NuPAGE Sample Reducing Agent (Life Technologies, Carlsbad, CA, USA) and distilled water were added to lysates. The samples were heated to 70°C for 10 min, and equal amounts of protein were loaded into a NuPAGE Novex 4–12% Bis-Tris gel (Life Technologies). Gel electrophoresis and blotting onto a PVDF membrane (Life Technologies) were performed according to the NuPAGE Technical Guide (Invitrogen). Tris buffered saline with 0.1% Tween-20 (Sigma Aldrich) and 5% Bio-Rad Blotting-grade blocker (Bio-Rad, Hercules, CA, USA) were used for blocking, while primary and secondary antibodies were diluted in the blocking buffer. Membranes were probed with antibodies against FPR1 and β-actin (both from Abcam, Cambridge, UK) and concentrations of the antibodies were 1:500 and 1:5000 respectively. Goat Anti-Rabbit IgG H&L (HRP) antibodies (Abcam, Cambridge, UK) at a 1:5000 dilution were used as secondary antibodies. SuperSignal West Pico Chemiluminescent Substrate (Pierce-Thermo scientific, Rockford, IL) was used for detection, and images were acquired on a Fujifilm LAS-3000 Imager.

## Results

### Anatomical distribution of intravenously injected ^125^I-fNLPNTL

**Fig 2:**
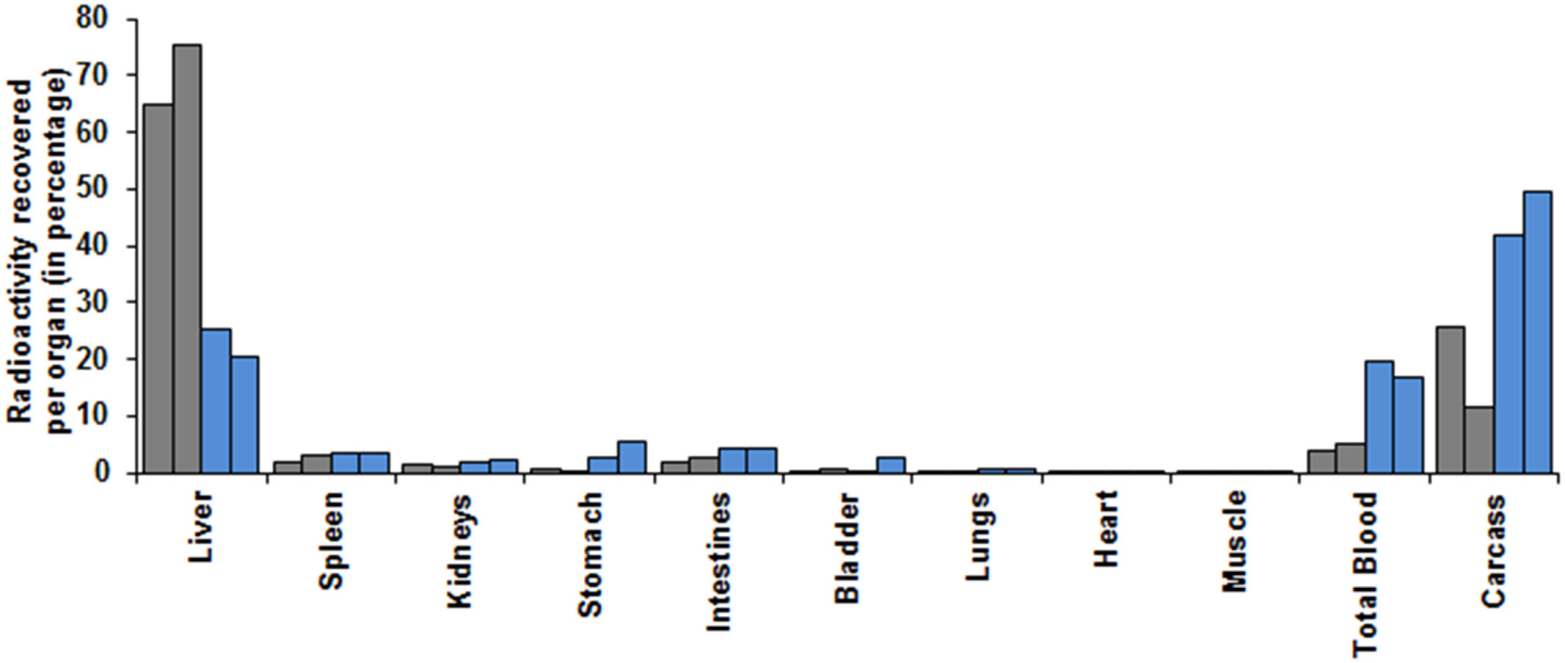
Anatomical distribution of i.v. administered fNLPNTL. The anatomical distribution of radio-labeled peptides was performed in C57BL/6 mice dosed i.v. with 8 μg of ^125^I-FITC-fNLPNTL (gray bars) or ^125^I-fNLPNTL (blue bars). Thirty min post-injection, the vasculature was washed free of blood and blood-borne radio-tracer by systemic perfusion through the heart. Blood, internal organs and carcass were harvested and analyzed for radioactivity. Data (n = 2 per group) represent the percentage of radioactivity counted in each organ, total blood and carcass, in relation to the total radioactivity recovered.

Native fNLPNTL and FITC-conjugated fNLPNTL (FITC- fNLPNTL) were labeled with ^125^I and intravenously injected into mice to assess their anatomical distribution. Analysis of organs 30 min post injection revealed that the liver was the major organ of uptake of both native and FITC- fNLPNTL (Fig 2). Of note, the uptake of ^125^I-FITC-fNLPNTL in the liver was three-fold higher compared to that of native fNLPNTL. This finding was accompanied by higher amounts of native fNLPNTL in the blood and rest of the carcass, thus suggesting a slower blood clearance of the native fNLPNTL compared to FITC-fNLPNTL.

### Expression of FPR1 in liver tissue and cells

Formyl peptide receptor 1 (FPR1) is a broadly expressed receptor with pattern recognition properties involved in diverse biological events. To investigate wether the uptake of the formyl peptide in the liver was mediated by FPR1, we determined the expression of FPR1 in isolated liver cells and tissue. In addition, we challenged mouse and human liver cell cultures with FITC-fNLPNTL and assessed the uptake by fluorescence microscopy.

**Fig 3:**
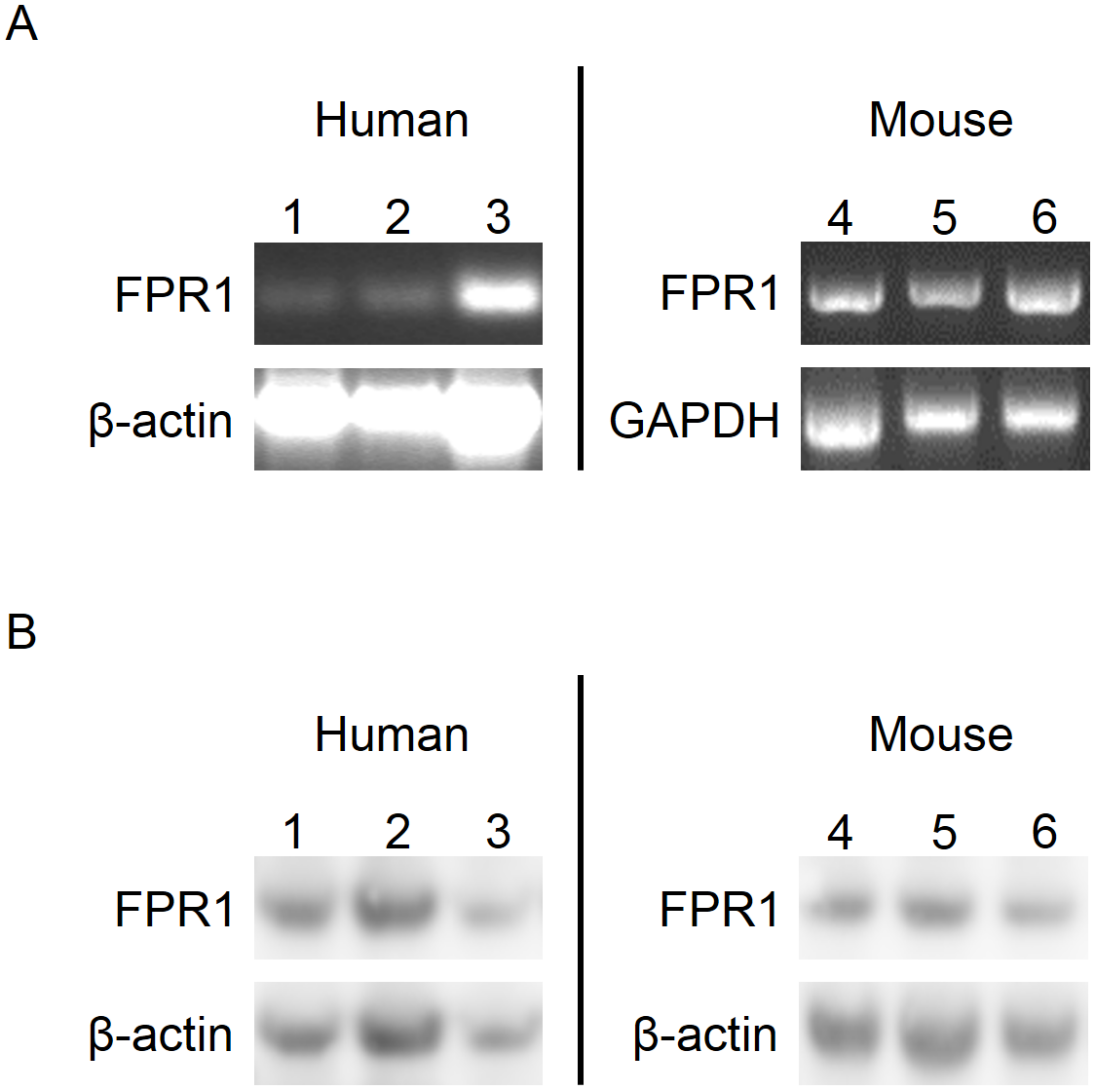
Expression of FPR1 mRNA and protein in liver and isolated liver cells. (A) Total RNA was isolated from pelleted cells and whole tissue. cDNA was synthesized from 1 μg of total RNA using the QuantiTect Reverse Transcription Kit. PCR was performed in a 25 μl reaction mixture containing 2 μl of cDNA of hHeps (1), hLSECs (2), human liver (3), mHeps (4), mLSECs (5), and mouse liver (6). The house keeping genes were β-actin and GAPDH. (B) Homogenized tissue specimens and cells were lysed in a RIPA lysis buffer with protease and phosphatase inhibitor cocktails. Equal amounts of protein were loaded into NuPAGE Novex 4–12% Bis-Tris gel, then blotted onto PVDF membrane and probed with antibodies against FPR1 and β-actin.

FPR1 expression in LSECs was performed by RT-PCR analysis using specific primers for murine and human FPR1. The cDNA detected by gel electrophoresis indicates the presence of FPR1 transcripts in both human and mouse Heps and LSECs (Fig 3A). Stabilin 2 and CRP were used as LSEC and Heps specific markers, respectively (not shown). Western blot analyses of tissue and cell lysates confirmed the PCR results (Fig 3B).

**Fig 4:**
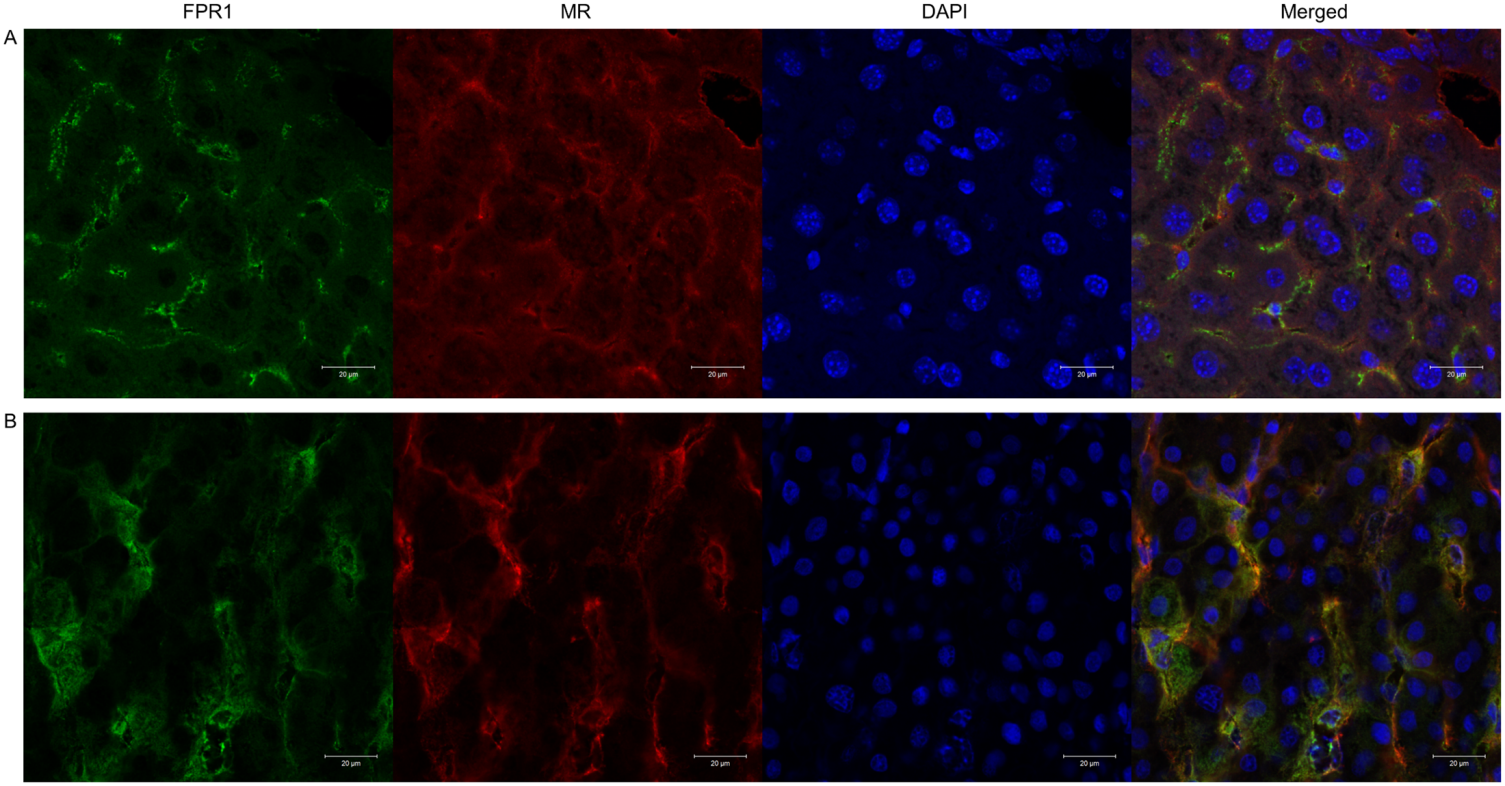
Immunohistochemical analysis of expression of FPR1 in human and mouse liver tissue. Liver sections were fixed with cold acetone for 10 min, washed in PBS and incubated overnight at 4°C with polyclonal rabbit anti-FPR1 (1:200), followed by Alexa Fluor^®^-488 goat anti-rabbit (1:1000). Intense FPR1 staining can be observed along the sinusoids in mouse liver (A), while in human liver the staining was more evenly localized to both LSECs and Heps (B). Double staining was performed using polyclonal rabbit anti-FPR1, rat anti-mouse CD206, and goat anti-human CD206, followed by Alexa Fluor^®^-488 and Alexa Fluor^®^-594 secondary antibodies, respectively.

**Fig 5:**
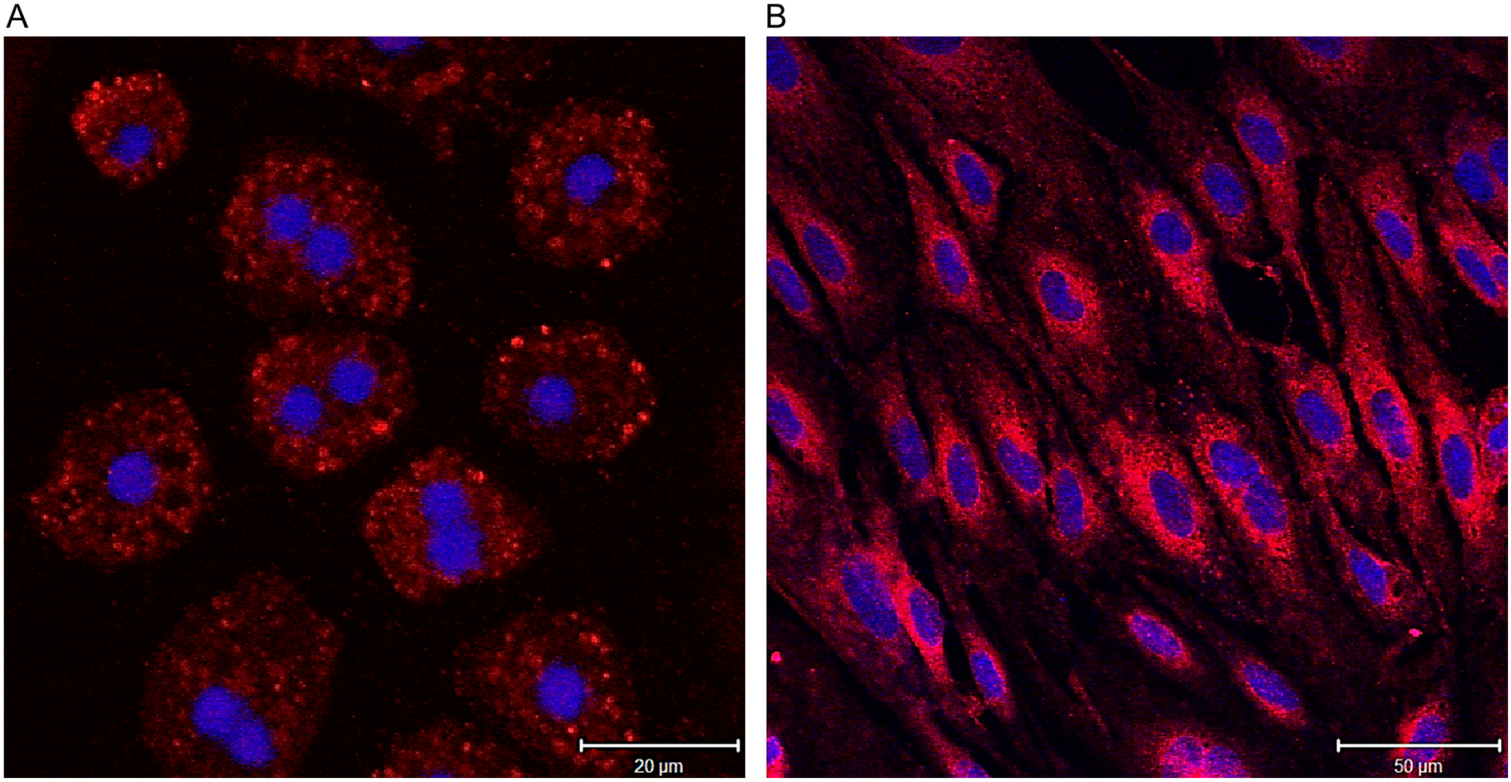
Immunocytochemical analysis of expression of FPR1 in isolated human liver cells. Primary cultures of hHeps (A) and LSECs (B) fixed with 4% PFA were stained for FPR1 using polyclonal rabbit anti-FPR1 (1:200) and Alexa Fluor^®^-532goat anti-rabbit (1:1000). Isotype IgGs (same concentration as primary antibodies), or secondary antibodies only were used as negative controls. Nuclei were stained with Draq5 in PBS (1:1000). Zeiss LSM 780 confocal laser scanning microscope was used for picture acquisition.

Immunohistochemistry of human and mouse liver tissue, and on isolated Heps and LSECs, revealed positive staining for FPR1. In murine liver, the staining was mainly observed in the cells lining the sinusoids (Fig 4A), while in human liver the staining was more evenly localized to both sinusoidal cells and Heps (Fig 4B). Double immunofluorescence staining using an anti-FPR1 antibody and an anti-mannose receptor (CD206:MR) antibody, an LSEC marker [20], demonstrated that the FPR1 is expressed by LSECs. FPR1 was also detected on isolated hHeps and hLSECs in primary cultures, as shown by immunofluorescence (Fig 5).

### Characterization of binding specificity

**Fig 6:**
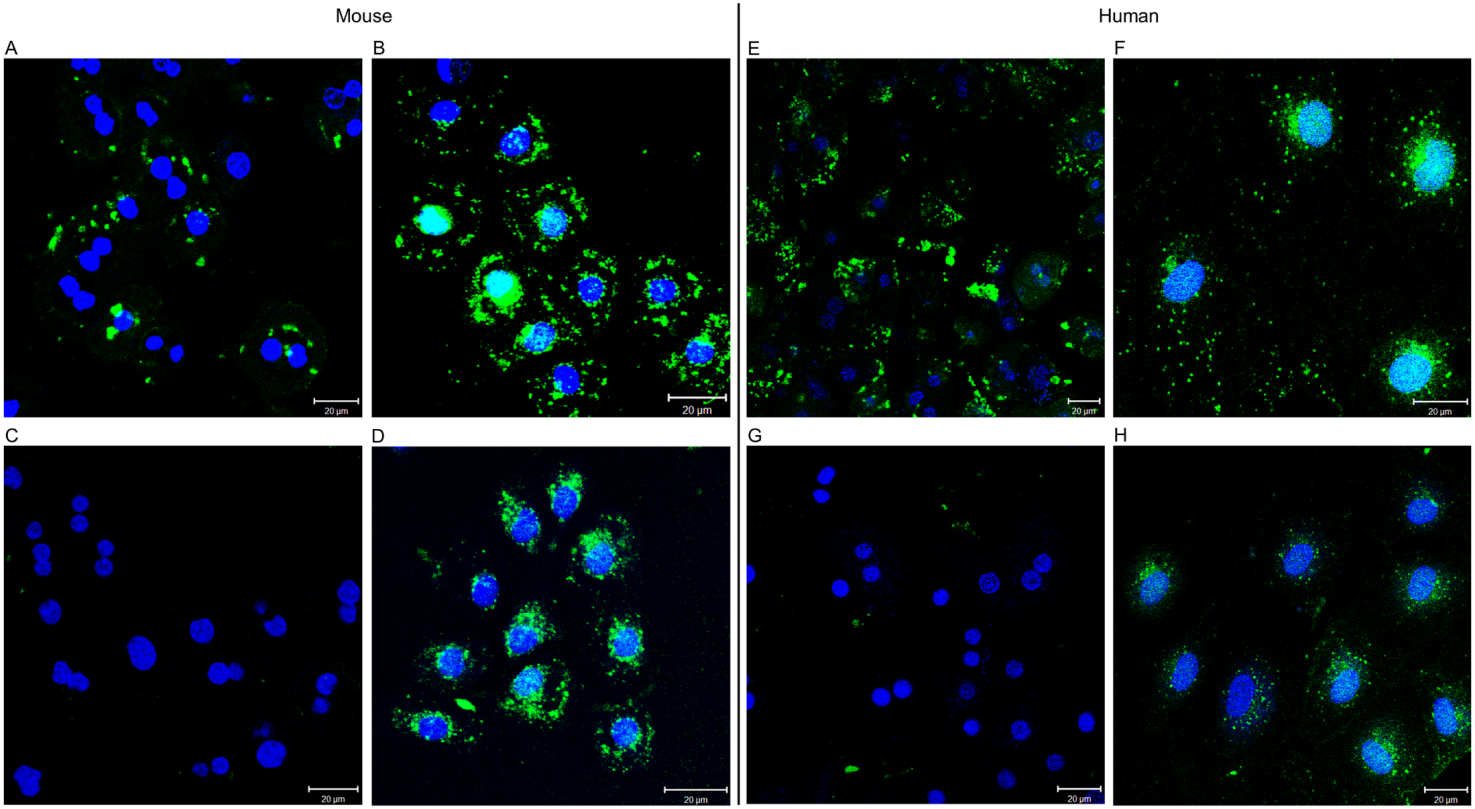
Ability of native fNLPNTL to compete with FITC-fNLPNTL for uptake in cultured murine and human LSECs and hepatocytes. Cultures of mHeps (A), mLSECs (B), hHeps (E), and hLSECs (F) were serum starved and incubated for 60 min at 37°C with 20 μg/ml of FITC-fNLPNTL in the presence or absence of 100 μg/ml of native fNLPNTL. After the removal of unbound ligands by extensive washing with PBS, the cultures were fixed with 4% PFA and processed for fluorescence microscopy. Uptake of FITC-fNLPNTL in mHeps (C) and hHeps (G) was blocked by native fNLPNTL, while a less marked effect was seen in mLSECs (D) and hLSECs (H).

The involvement of FPR1 in the uptake of formyl peptide in different human and murine liver cells was investigated by incubating cultured LSECs and Heps with trace amounts of FITC-fNLPNTL in the presence or absence of excess concentrations of native fNLPNTL (Fig 6). We found that the inhibitory effect of native peptide was modest in LSECs, but apparently complete in Heps, suggesting that Heps took up FITC-fNLPNTL by the same mechanism as unconjugated fNLPNTL, whereas the uptake of FITC-fNLPNTL could be only partially inhibited by excess fNLPNTL.

**Fig 7:**
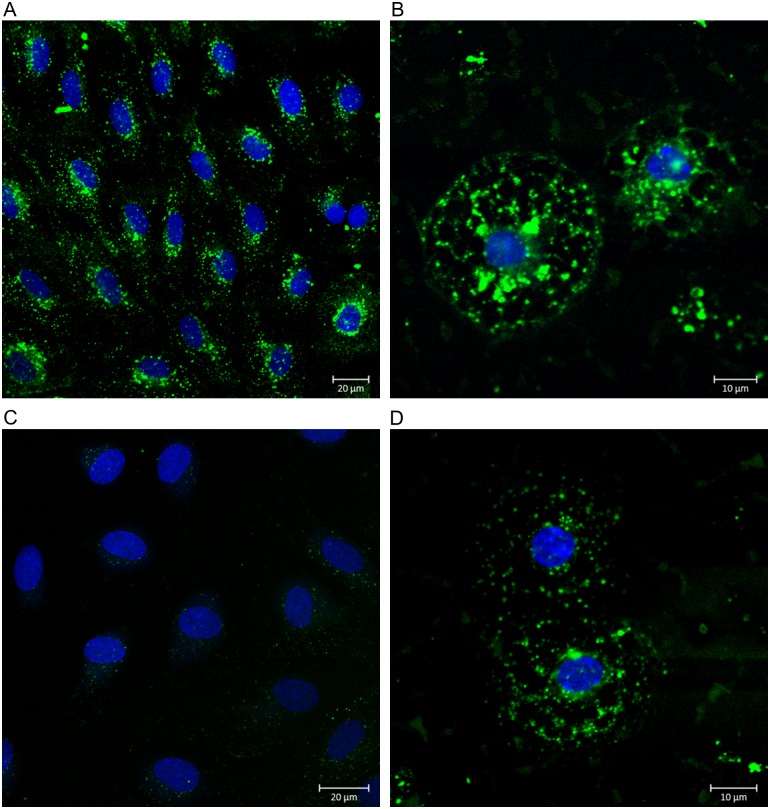
Competitive effect of FSA on FITC-fNLPNTL uptake by human and murine LSECs. Primary cultures of hLSECs (A) and mLSECs (B) were established on Permanox chamber slides coated with human fibronectin. Following 4 h of serum-free starvation, the cells were incubated for 60 min at 37°C with 20 μg/ml of FITC-fNLPNTL in a serum-free culture medium, in the presence or absence of 100 μg/ml of FSA. After removal of unbound ligands by extensive washing with PBS, the cultures were fixed with 4% PFA and processed for fluorescence microscopy. Uptake of FITC-fNLPNTL was inhibited by the presence of FSA in hLSECs (C) to a higher extent than seen in mLSECs (D).

To further investigate this we incubated LSECs with FITC-fNLPNTL only, or in the presence of formaldehyde-treated-serum-albumin (FSA), a ligand known to be specifically endocytosed by the LSECs scavenger receptors (Fig 7). These studies revealed that the uptake of FITC-fNLPNTL was inhibited by the presence of FSA in both human and murine LSECs, although to a higher extent in hLSECs (Fig 7C and 7D). This difference may be due to the much higher endocytic activity of freshly isolated mLSECs compared to hLSECs, which have been cultured for five days prior to the experiment.

## Discussion

The liver sinusoidal endothelial cell (LSEC) represents a unique class of endothelial cells distinct from the other classes of endothelial cells in the body [21]. Functionally, this cell type has an endocytic machinery capable of very efficient uptake and degradation of physiological and foreign waste material, including all major classes of biological macromolecules and nanoparticles (< 200 nm) [22]. The endocytic capacity of LSECs greatly exceeds that of other types of endothelial cells. The signature feature of LSECs as scavenger cells, responsible for the removal of potentially dangerous macromolecules from blood, emphasizes their importance in liver immunity.

Formyl peptides labeled with FITC or other fluorochromes are increasingly used to identify cells involved in inflammatory reactions since it is generally believed that this probe, when administered i.v., will effectively target cells that carry the receptor for formyl peptides, FPR1, in the same way as unconjugated fNLPNTL. We found it important to verify this hypothesis, and devised a series of experiments to study whether FITC-fNLPNTL exhibits the same *in vivo* distribution pattern as fNLPNTL. In order to trace the injected ligand, the fNLPNTL was radioiodinated by two distinct features, through labeling the FITC group of the FITC-fNLPNTL conjugate or directly to a tyrosine group within fNLPNTL.

First, we investigated the fate of fNLPNTL administered i.v. into mice. Interestingly, FITC-fNLPNTL was taken up in the liver to a much greater extent than was fNLPNTL. Moreover, the unconjugated peptide distributed to blood and carcass to a much higher extent than its FITC-labeled counterpart (Fig 2).

We next wanted to identify the receptors involved in the uptake of the conjugated and unconjugated peptides. First, we established that FPR1, on both mRNA and protein basis was present in the liver and isolated liver cells of mice and humans. Subsequent immunohistochemistry on mouse- and human liver sections showed that FPR1 was present in LSECs and Heps, with a higher expression in mouse LSECs. The coincident staining of mannose receptor and FPR1 in sinusoidal cells suggested that the receptor protein was expressed in LSECs. Immunocytochemistry on isolated human liver cells showed the expression of FPR1 in both LSECs and Heps. The finding that excess unconjugated fNLPNTL blocked the uptake of FITC-fNLPNTL in mouse and human Heps showed that these cells did not distinguish between the two ligands. This is a strong indication that conjugation with FITC does not change the recognition of the peptide in Heps. The same type of ligand-receptor competition experiments in isolated LSECs showed that excess unconjugated fNLPNTL only partly inhibited the uptake of FITC-fNLPNTL. We interpret this to mean that LSECs use at least one more type of receptors in addition to FPR1 in their uptake of FITC-fNLPNTL. Hence, the dramatic increase in vivo of hepatic clearance of fNLPNTL after FITC conjugation is attributable to increased LSECs uptake. To determine whether this other receptor might be the “work horse” scavenger receptor of LSECs, namely the LSEC-specific scavenger receptor, or stabilin 1/2, we incubated isolated murine and human LSECs with FITC-fNLPNTL in the presence or absence of formaldehyde-modified serum albumin (FSA), a ligand that is frequently used to study specific stabilin-mediated endocytosis in LSECs [23]. The observation that FSA inhibited the uptake of FITC-fNLPNTL in both human and mouse LSECs strongly suggests that these scavenger cells use stabilin to clear FITC-fNLPNTL from the circulation.

It has been previously reported that FITC labeling of proteins drastically shorten their serum half-lives, causing their rapid clearance in the liver [16]. On this background we hypothesized that FITC labeling may have the same liver targeting effect on fNLPNTL. An increased net negative charge of proteins resulting from occupying positively charged lysyl residues by conjugation with fluorescein via the isothiocyanate group leads to an increased uptake in LSECs [24]. In conclusion, our finding emphasizes the role of LSECs as important neutralizers of otherwise strong inflammatory signals such as formyl peptides associated with both DAMPS and PAMPS.

Importantly, conjugated flurochromes such as FITC-fNLPNTL must be used with great caution in systemic studies on the biodistribution of fNLPNTL since a considerable proportion of an injected dose of FITC-fNLPNTL will end up in the powerful LSEC scavenger cells through a mechanism that is caused by FITC conjugation. This could give a false impression of presence and location of inflammatory cells in the liver.

## Supporting Information

S1 Fig Complete WB membranes and PCR gels images for Fig 3. (A) Complete PCR gel representing human data for Fig 3. Lanes 1, 2 and 3 correspond to hHeps, hLSECs and human liver respectively. Lanes 4 are no template controls. (B) Complete PCR gel representing mouse data for Fig 3. Lanes 1, 2 and 3 correspond to mHeps, mLSECs and mouse liver respectively. Lanes 4 are no template controls. (C) WB membrane representing human data for Fig 3. Lanes 1, 2 and 3 correspond to hHeps, hLSECs and human liver respectively. (D) WB membrane representing mouse data for Fig 3. Lanes 1, 2 and 3 correspond to mHeps, mLSECs and mouse liver respectively.
(TIF)
