# Supplementary figures and images for: FITC Conjugation Markedly Enhances Hepatic Clearance of N-Formyl Peptides
Source: PLoS One. 2016 Aug 5;11(8):e0160602. doi: 10.1371/journal.pone.0160602 (PMC4975464; doi:10.1371/journal.pone.0160602)

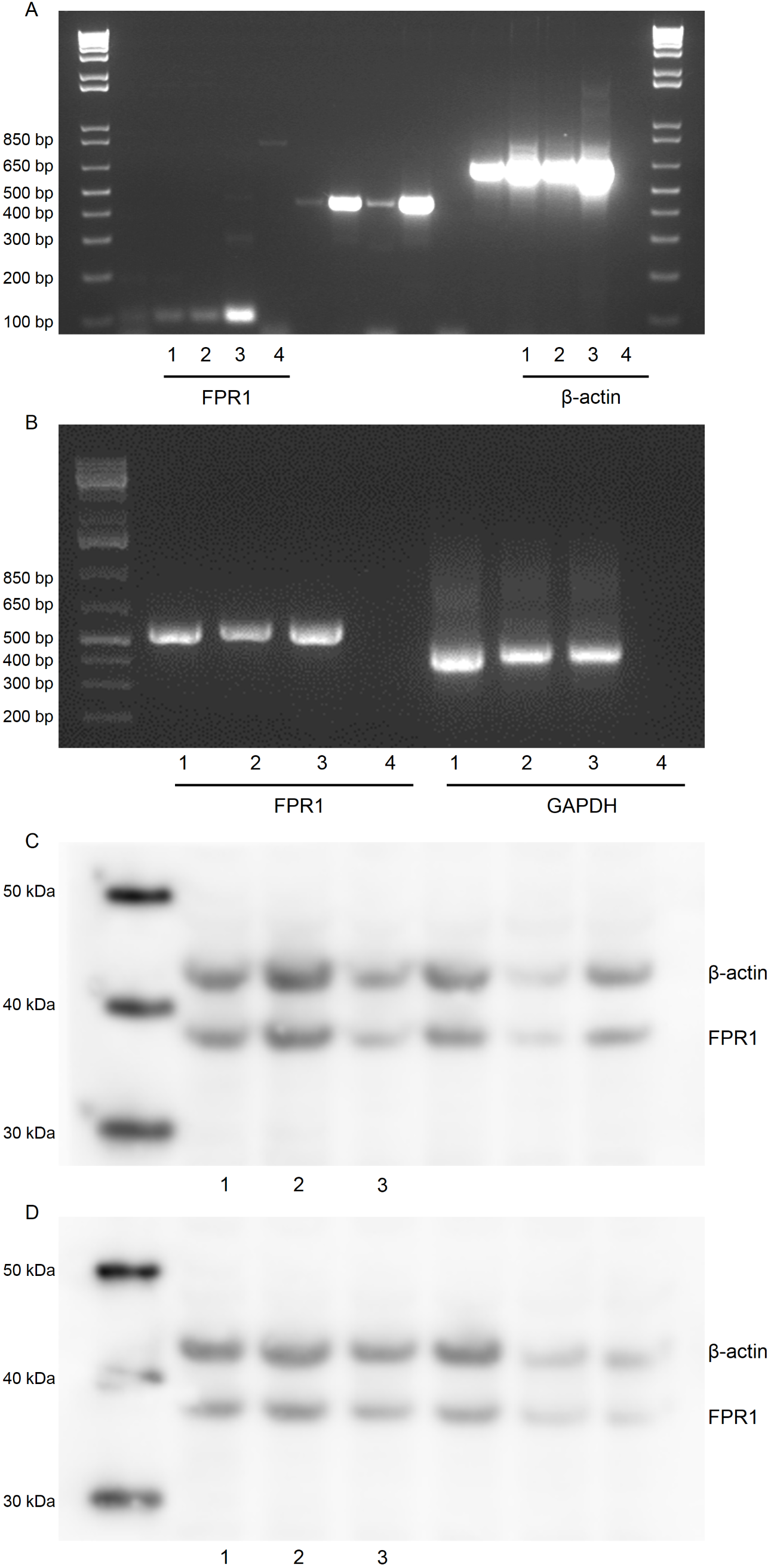

Supplement: S1 Fig — (A) Complete PCR gel representing human data for Fig 3. Lanes 1, 2 and 3 correspond to hHeps, hLSECs and human liver respectively. Lanes 4 are no template controls. (B) Complete PCR gel representing mouse data for Fig 3. Lanes 1, 2 and 3 correspond to mHeps, mLSECs and mouse liver respectively. Lanes 4 are no template controls. (C) WB membrane representing human data for Fig 3. Lanes 1, 2 and 3 correspond to hHeps, hLSECs and human liver respectively. (D) WB membrane representing mouse data for Fig 3. Lanes 1, 2 and 3 correspond to mHeps, mLSECs and mouse liver respectively. (TIF) [file pone.0160602.s001.tif]
